# Bilateral Optic Sheath Enhancement in Giant Cell Arteritis (GCA) Presenting With Occipital Neuralgia-Like Symptoms

**DOI:** 10.7759/cureus.98136

**Published:** 2025-11-30

**Authors:** Ahmed Malik Abuelgasim Malik, Hossam Malik Abdalla, Yun Zou, Thomas Lambert

**Affiliations:** 1 Acute Internal Medicine, University Hospitals of North Midlands, Royal Stoke University Hospital, Stoke-on-Trent, GBR; 2 Neurology, University Hospitals of North Midlands, Royal Stoke University Hospital, Stoke-on-Trent, GBR; 3 Rheumatology, Midlands Partnership NHS Foundation Trust, Stoke-on-Trent, GBR

**Keywords:** angiotensin converting enzyme (ace), anterior ischemic optic neuropathy (aion), antineutrophil cytoplasmic antibody (anca), antinuclear antibodies (ana), computed tomography (ct), c-reactive protein (crp), erythrocyte sedimentation rate (esr), giant cell arteritis (gca), myelin oligodendrocyte glycoprotein antibody-associated disease (mogad), neuromyelitis optica spectrum disorders (nmosd)

## Abstract

We present the case of an 86-year-old woman admitted with a two-week history of a bilateral occipital burning sensation, without fever, jaw claudication, or neck stiffness. Laboratory investigations revealed a markedly elevated C-reactive protein (CRP) level of 284 mg/L. Magnetic resonance imaging (MRI) of the head with contrast demonstrated bilateral hyperenhancement of the optic nerve sheaths. Although optic nerve signal changes are an uncommon manifestation of giant cell arteritis (GCA), the neurology team commenced high-dose oral prednisolone (60 mg once daily), considering GCA in the differential diagnosis. The patient showed rapid symptomatic and biochemical improvement following steroid initiation. Subsequent temporal artery ultrasound findings were consistent with GCA, supporting the diagnosis. This case highlights an unusual radiological presentation of GCA and underscores the importance of early empirical treatment to prevent irreversible visual complications.

## Introduction

Giant cell arteritis (GCA) is a chronic granulomatous vasculitis affecting large- and medium-sized arteries, typically occurring in patients aged ≥50 years and frequently associated with polymyalgia rheumatica [1,2]. Classical clinical manifestations include headache, scalp tenderness, jaw claudication, reduced temporal artery pulsation, and visual symptoms such as diplopia and amaurosis fugax [1,3]. Laboratory evaluation usually demonstrates markedly elevated inflammatory markers, including erythrocyte sedimentation rate (ESR) and C-reactive protein (CRP) [1,4,5]. Without prompt initiation of high-dose glucocorticoid therapy, GCA may result in occlusion of cranial arteries, leading to irreversible complications such as blindness or stroke. These events most often occur before treatment or within the first week of therapy, making GCA a true medical emergency [6-8].

Neuro-ophthalmic manifestations, such as anterior ischemic optic neuropathy (AION), are well recognised in GCA; however, optic nerve sheath enhancement on magnetic resonance imaging (MRI) is a rare finding, with only a handful of cases described in the literature [2,3-6]. Isolated occipital neuralgia as a presenting symptom is also uncommon [7,9]. Our case is notable for the combination of atypical occipital neurological symptoms and bilateral optic nerve sheath enhancement on MRI, which broadened the initial differential diagnosis to include neuromyelitis optica spectrum disorders (NMOSD) and myelin oligodendrocyte glycoprotein antibody-associated disease (MOGAD) [9]. This report underscores the importance of recognising atypical radiological and clinical features of GCA to facilitate early treatment and prevent irreversible visual loss.

## Case presentation

An 86-year-old woman presented with a 10-day history of bilateral burning paraesthesia over the occipital region, radiating to the temples and forehead. The symptoms were most pronounced at 4:00 a.m., with an intensity of 8/10, and were accompanied by scalp sensitivity and headache, but no vomiting. She also reported a seven-day history of night sweats and fatigue. She denied visual symptoms, dysarthria, dysphagia, focal weakness, sensory loss, rash, constitutional ENT symptoms, or features suggestive of peripheral neuropathy. Her past medical history included hypertension, dyslipidaemia, diet-controlled type 2 diabetes mellitus, chronic kidney disease, and gallstones. She had a family history of rheumatoid arthritis and polymyalgia rheumatica in her sister. Medications included amlodipine and simvastatin. She was a non-smoker and consumed alcohol occasionally. On examination, cranial nerves were intact, with full extraocular movements, and no ptosis, diplopia, or nystagmus. Motor power, coordination, reflexes, and plantar responses were normal. Sensation was preserved, and tandem gait was intact. Fundoscopy revealed normal optic discs bilaterally. Given the suspicion for GCA based on clinical presentation and markedly elevated inflammatory markers, she was commenced on oral prednisolone 60 mg once daily [1,8]. Within 48 hours, her symptoms resolved completely. Ophthalmological review confirmed normal optic discs bilaterally with no signs of AION [3,6]. Rheumatological examination revealed thickening over the left temporal artery. Both temporal arteries were non-tender with palpable pulses, and peripheral pulses were normal. No carotid bruits, scalp tenderness, or proximal muscle weakness were noted. She was placed on a 12-18-month prednisolone taper, with gastroprotective (omeprazole) and bone-protective therapy (calcium/vitamin D supplementation and alendronic acid). The laboratory blood testing is shown in Table 1 [1,8].

**Table 1:** Laboratory Findings ESR: erythrocyte sedimentation rate, CRP: C-reactive protein, ACE: angiotensin-converting enzyme, ANCA: anti-neutrophil cytoplasmic antibody, ENA: extractable nuclear antigen, NMOSD: neuromyelitis optica spectrum disorders, MOGAD: myelin oligodendrocyte glycoprotein antibody-associated disease, SLE: systemic lupus erythematosus

Test	Result	Reference Range	Interpretation
ESR	27 mm/hr	< 20 mm/hr	Mildly elevated
CRP	284 mg/L	< 5 mg/L	Markedly elevated
ESR (after 2 weeks steroids)	Normalized	< 20 mm/hr	Returned to normal
CRP (after 2 weeks steroids)	Normalized	< 5 mg/L	Returned to normal
Serum ACE	Normal	8–65 U/L	Within normal range
Lyme serology	Negative	Negative	No evidence of infection
Syphilis serology	Negative	Negative	No evidence of infection
HIV	Negative	Negative	No evidence of infection
Paraneoplastic panel	Negative	Negative	No evidence of paraneoplastic disease
Aquaporin-4 antibodies	Negative	Negative	No evidence of NMOSD
Anti-MOG antibodies	Negative	Negative	No evidence of MOGAD
ANCA	Negative	Negative	No evidence of vasculitis
Anti-dsDNA	Negative	Negative	No evidence of SLE
ENA panel	Negative	Negative	No evidence of connective tissue disease

**Figure 1:**
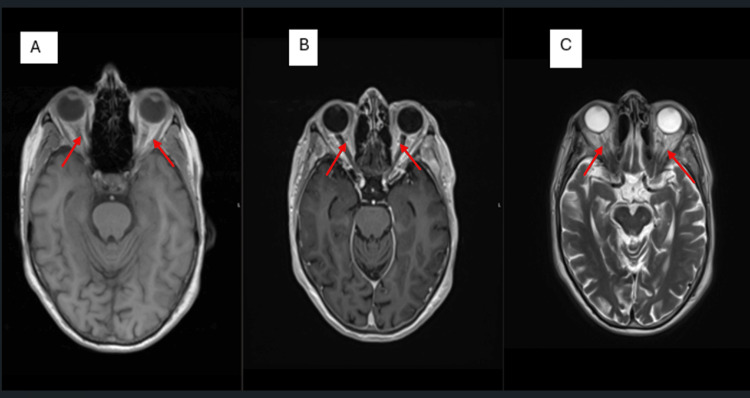
Magnetic resonance imaging (MRI) head showing bilateral optic nerve signal changes. A: precontrast MRI T1, B: postcontrast MRI T1 and C: MRI fluid-attenuated inversion recovery (FLAIR) T2

Magnetic resonance imaging (MRI) of the head performed prior to steroid initiation demonstrated bilateral optic nerve sheath hyperenhancement on contrast-enhanced T1-weighted imaging (Figure 1). This finding, while rarely reported in GCA [3-6,9], broadened the differential diagnosis to include NMOSD and MOGAD [2].

**Figure 2:**
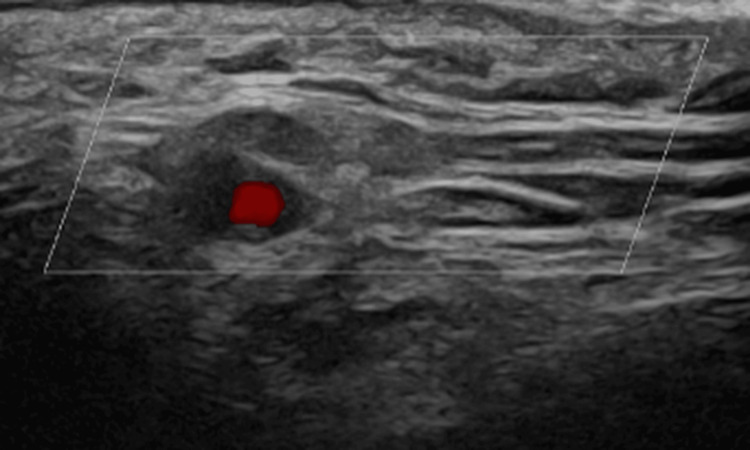
Ultrasound temporal artery scan. Halo sign in left common temporal artery, in keeping with giant cell arteritis (GCA)

A temporal artery ultrasound performed after four doses of prednisolone demonstrated non-compressible, hypoechoic thickening of the righ temporal common and frontal branches and the left common, frontal, and parietal branches, consistent with the “halo sign” (Figure 2) [1,8]. Computed tomography (CT) of the thorax, abdomen, and pelvis revealed no evidence of malignancy.

Treatment and outcome

The patient was commenced on oral prednisolone 60 mg once daily, with a tapering regimen initiated thereafter. She was co-prescribed omeprazole 40 mg once daily for gastric protection, alongside bone protection with Adcal-D3 and alendronic acid 70 mg once weekly. Remarkably, all her symptoms resolved completely within 48 hours of corticosteroid initiation. Inflammatory markers normalised, with both CRP and ESR returning to reference ranges within two weeks of treatment.

## Discussion

Only a limited number of GCA cases have been reported with MRI evidence of enhancement involving the orbital fat, optic nerve, or optic nerve sheath [2-4]. These MRI changes are thought to result from breakdown of the blood-brain barrier, a phenomenon seen in infiltrative, inflammatory, demyelinating, infectious, or neoplastic conditions, and are typically associated with visual symptoms and non-arteritic AION [3]. In our case, optic nerve sheath enhancement was present in the absence of AION, which is an uncommon finding. The most plausible mechanism is granulomatous inflammation similar to that affecting the temporal arteries, leading to disruption of the blood-optic nerve barrier [3,4,6]. An additional noteworthy feature in this case was the occipital neuralgia-like presentation, which has been documented previously in GCA and is likely attributable to involvement of the occipital arteries [7]. Recognition of such atypical symptom patterns is important, as they can delay diagnosis if not considered early. If optic nerve sheath enhancement is not recognised as a potential manifestation of GCA, it may be mistaken for other inflammatory conditions such as sarcoidosis, NMOSD, MOGAD, or granulomatosis with polyangiitis [2,5,6]. The concurrent finding of fusiform enlargement of the temporal arteries on imaging can reduce diagnostic uncertainty [5,6]. While MRI is not routinely indicated as a first-line diagnostic tool for GCA, it can be valuable in situations where imaging has already been performed for other reasons, or when diagnostic ambiguity exists. Enlargement or mural enhancement of the temporal arteries on MRI has been shown to have a sensitivity of 80.6% and a specificity of 97% for GCA [5,9]. Furthermore, these imaging findings may help guide the optimal site for temporal artery biopsy [5,9].

## Conclusions

Although GCA is primarily diagnosed through clinical assessment supported by laboratory markers and vascular ultrasound, MRI can provide valuable adjunctive information in selected scenarios. This is particularly relevant when the presentation is atypical, when visual symptoms such as papilloedema, visual impairment, or features suggestive of AION are present, or when the diagnosis remains uncertain despite standard investigations. In our case, MRI revealed bilateral optic nerve sheath enhancement - an uncommon but clinically significant finding - which broadened the differential diagnosis to include other inflammatory and demyelinating disorders. While MRI is not recommended as a first-line investigation for suspected GCA, high-resolution 3T MRI of the cranial arteries may be a useful alternative when temporal artery ultrasound or biopsy is not feasible, or when additional structural detail is required. Importantly, mural enhancement or enlargement of temporal arteries on MRI can further support the diagnosis and may help guide biopsy site selection. The most critical learning point from this case is the value of early empirical glucocorticoid therapy in suspected GCA, even when the presentation is atypical. Prompt initiation of steroids can prevent irreversible complications, including permanent vision loss, and should be considered a priority in the presence of supportive clinical and investigative findings. This case underscores the need for clinicians to maintain a high index of suspicion for GCA in patients with atypical cranial or neuro-ophthalmic symptoms, and to incorporate advanced imaging judiciously when conventional diagnostic pathways leave uncertainty.
